# Induction of p38- and gC1qR-dependent IL-8 expression in pulmonary fibroblasts by soluble hepatitis C core protein

**DOI:** 10.1186/1465-9921-6-105

**Published:** 2005-09-15

**Authors:** Jonathan P Moorman, S Matthew Fitzgerald, Deborah C Prayther, Steven A Lee, David S Chi, Guha Krishnaswamy

**Affiliations:** 1 Department of Internal Medicine, James H. Quillen College of Medicine, East Tennessee State University, Johnson City, TN, USA; 2 Medical Service, James H. Quillen VAMC, Johnson City, TN, USA

## Abstract

**Background:**

Recent studies suggest that HCV infection is associated with progressive declines in pulmonary function in patients with underlying pulmonary diseases such as asthma and chronic obstructive pulmonary disease. Few molecular studies have addressed the inflammatory aspects of HCV-associated pulmonary disease. Because IL-8 plays a fundamental role in reactive airway diseases, we examined IL-8 signaling in normal human lung fibroblasts (NHLF) in response to the HCV nucleocapsid core protein, a viral antigen shown to modulate intracellular signaling pathways involved in cell proliferation, apoptosis and inflammation.

**Methods:**

NHLF were treated with HCV core protein and assayed for IL-8 expression, phosphorylation of the p38 MAPK pathway, and for the effect of p38 inhibition.

**Results:**

Our studies demonstrate that soluble HCV core protein induces significant increases in both IL-8 mRNA and protein expression in a dose- and time-dependent manner. Treatment with HCV core led to phosphorylation of p38 MAPK, and expression of IL-8 was dependent upon p38 activation. Using TNFα as a co-stimulant, we observed additive increases in IL-8 expression. HCV core-mediated expression of IL-8 was inhibited by blocking gC1qR, a known receptor for soluble HCV core linked to MAPK signaling.

**Conclusion:**

These studies suggest that HCV core protein can lead to enhanced p38- and gC1qR-dependent IL-8 expression. Such a pro-inflammatory role may contribute to the progressive deterioration in pulmonary function recently recognized in individuals chronically infected with HCV.

## Background

Hepatitis C virus (HCV), an RNA virus of the Flavivirus family, is the most common blood-borne infection in the United States [1,2]. A striking feature of HCV disease is the high rate of progression to chronicity, with over 80% of acutely infected individuals developing chronic inflammation [3]. This inflammation has been associated with liver failure, hepatocellular carcinoma and autoimmune dysfunction [1]. Treatment for HCV is toxic and of limited efficacy, and the majority of infected individuals do not receive the antiviral therapies available.

Recently, HCV infection has been repeatedly linked to progressive declines in pulmonary function in patients with underlying lung diseases such as asthma and chronic obstructive pulmonary disease (COPD) [4,5]. In patients who already had a diagnosis of COPD, chronic HCV infection led to a more rapid decline in forced expiratory volume (FEV1) and diffusing capacity for carbon monoxide (DLCO), findings that were abrogated in those treated with interferon [4]. In a recent 6-year prospective trial, asthmatic patients with chronic HCV who did not respond to interferon had greater impaired reversibility to bronchodilators when compared to either HCV-negative controls or to HCV-positive individuals who responded to interferon. [5] Some data suggests that HCV infection may alter acetylcholine-mediated airway tone [5]. Other smaller studies also suggest a role for HCV infection in various pulmonary diseases, including idiopathic pulmonary fibrosis [6,7].

While the pathogenesis of the progressive liver disease that occurs with HCV infection involves fibrosis of hepatic tissue in the setting of chronic inflammation, there are few data available that address the inflammatory aspects of HCV infection that lead to declines in lung function. Studies in chronically infected individuals have however demonstrated increased levels of both serum and intrahepatic cytokines, in particular interleukin-8 (IL-8), a chemokine well-known to mediate inflammatory pulmonary processes [8,9]. IL-8 is involved in host inflammatory responses and is synthesized by many different cell types, including fibroblasts and epithelial cells. Expression of IL-8 may inhibit the antiviral activity of interferon γ (IFN) [9] and correlates with the degree of hepatic fibrosis and portal inflammation during HCV infection [10,11]. While IL-8 plays a significant role in pulmonary pathology in general [12], its role in pulmonary disease specifically associated with HCV has not been addressed.

IL-8 signaling is characterized by the integration of at least three different signaling pathways that coordinate induction of mRNA synthesis or that suppress mRNA degradation [13]. Current models suggest that maximal IL-8 can be generated upon de-repression of the gene promoter, activation of NFκB and JNK pathways, and stabilization of the resulting mRNA by p38 MAPK signaling. ERK signaling also contributes to IL-8 induction, although it does not appear to be a potent inducer. TNFα likely activates all of these pathways and has served as a model for robust IL-8 signaling.

Interestingly, we and other investigators have found that the nucleocapsid core protein of HCV may modulate immune signaling pathways, including those mediated by receptors such as gC1qR, TNFR1, and Fas [14-16]. This protein has been found in serum in naked form [17], and soluble core protein can bind and signal extracellularly via the complement receptor, gC1q, on lymphocytes [15]. HCV core appears to be the most potent signal inducer of the IL-8 promoter in hepatocytes transfected with viral protein-reporter expression vectors [18].

We would like to better understand the mechanisms by which chronic HCV infection leads to a more progressive pulmonary decline in individuals with chronic lung disease. Because HCV core antigen can modulate immune signaling pathways that affect IL-8 transcription, we examined the role of soluble HCV core protein in IL-8 signaling in pulmonary fibroblasts. We document an HCV core-induced increase in IL-8 mRNA and protein expression in fibroblasts that is both dose- and time-dependent. We demonstrate that this increase is associated with activation of p38 MAPK that this activation is necessary. We show that co-signaling with TNFα and HCV core leads to augmentation of IL-8 gene and protein expression. Finally, we document that HCV core-mediated IL-8 up-regulation can be inhibited using antibodies that block gC1qR, a known receptor for soluble HCV core antigen.

## Materials and methods

### Tissue Culture

Normal human lung fibroblasts (NHLF) (Clonetics-BioWhittaker, Walkersville, MD) were grown in fibroblast basal medium (Clonetics-BioWhittaker, Walkersville, MD) at 5% CO_2 _at 37°C. Media was supplemented with 2% fetal bovine serum, human fibroblast growth factor-B (1.0 μg/mL), insulin (5 mg/mL), gentamicin and amphotericin B. NHLF were cultured in 12 well culture plates at a cell concentration of 5 × 10^4 ^cells per well and incubated overnight. β-galactosidase-HCV core antigen (1–191) fusion proteins (core) or β-galactosidase control proteins (β-gal) (endotoxin-negative; Virogen, Watertown, MA) were added at the indicated concentrations based on previous studies of soluble core antigen [15,19] and incubated for 24 hours. Time course experiments were done in the same manner for 12, 24, and 48 hours. Tumor necrosis factor α (TNFα) was added at 1 U/mL with and without HCV core antigen and incubated for 24 hours. Inhibition of p38 MAPK studies were done using the specific inhibitor, SB203580 (Calbiochem, San Diego, CA) as described [20]. NHLF were pretreated with SB203580 (10 μM) for two hours prior to addition of HCV core antigen or control proteins. Murine gC1qR-specific antibodies (Chemicon, Temicula, CA) were used at two different concentrations as described.

### Enzyme Linked Immunosorbent Assay (ELISA)

Enzyme linked immunosorbent assay (ELISA) was used to detect IL-8 levels in cell-free supernatants as previously described using commercially available kits (R&D Systems, Minneapolis, MN) [20]. Values were extrapolated or interpolated from a standard curve. Results were analyzed on an ELISA plate reader (Dynatech MR 5000 with supporting software).

### IL-8 Gene Expression by RT-PCR

Gene expression for IL-8 was assessed using RT-PCR as previously described [21]. RNA was extracted by a RNAzol technique from cultured cells. Briefly, total cellular RNA was extracted from cultured cells (1 × 10^6 ^cells) by the addition of 1.1 mL of RNAzol B (Tel-Test, Inc., Friendswood, Texas). The suspension was shaken for 1 minute and centrifuged at 12,000 × g for 15 minutes at 4°C. The aqueous phase was washed twice with 0.8 ml phenol:chloroform (1:1, v/v), and once with 0.8 mL of chloroform. Each time, the suspension was centrifuged at 12,000 × g for 15 minutes at 4°C. An equal volume of isopropanol was added to the aqueous phase, and the preparation refrigerated at -20°C overnight. After centrifugation at 12,000 × g for 30 minutes at 4°C, the RNA pellet was washed with 75% ethanol. The RNA pellet was air dried and suspended in 20 μl of DEPC-treated water. RNA was quantitated by optical density readings at 260 nm, and the integrity of the 28S and 18S RNA bands determined by electrophoresis in ethidium bromide-stained agarose gels. First strand cDNA was synthesized in the presence of murine leukemia virus reverse transcriptase (2.5 U/μL), 1 mM each of the nucleotides dATP, dCTP, dGTP and dTTP; RNase inhibitor (1 U/μL), 10× PCR buffer (500 mM KCl, 100 mm Tris-HCl, pH 8.3), and MgCl_2 _(5 mM), using oligo(dT)_16 _(2.5 mM) as a primer. The preparation was incubated at 42°C for 20 minutes in a DNA thermocycler (Perkin-Elmer Corp., Norwalk, CT) for reverse transcription. PCR amplification was done on aliquots of the cDNA in the presence of MgCl_2 _(1.8 mM), dNTPs (0.2 mM), and AmpliTaq polymerase (1 U/50 μL), and paired cytokine-specific primers (0.2 nM of each primer) to a total volume of 50 μl. Paired primers for the housekeeping gene hypoxanthine phosphoribosyltransferase (HPRT) were employed as a control for gene expression. PCR consisted of 1 cycle of 95°C for 2 min, 45 cycles of 95°C for 45 sec, 60°C for 45 sec, and 72°C for 1 min 30 sec, and lastly, 1 cycle of 72°C for 10 min. Fourteen microliters of the amplified products were subjected to electrophoresis on a 2% agarose gel stained with ethidium bromide. IL-8 gene products were confirmed by fragment size.

### Immunofluorescent Staining

NHLF were cultured on sterile coverslips overnight and subsequently treated with β-gal or core (1 μg/ml) for 30 minutes to three hours. Cells were fixed by immersion in ice-cold methenol:acetone (1:1) for ten minutes at 20°C. Coverslips were air-dried and cells blocked with 1% normal donkey serum (Jackson Laboratories, West Grove, PA) in PBS for thirty minutes. A primary polyclonal rabbit antibody to phosphorylated p38 (Cell Signaling Tech, Beverly, MA) was diluted 1:200 in 1% normal donkey serum/PBS and incubated with cells for 1.5 hr at room temperature. Cells were washed three times using PBS with Tween-20 at five minute intervals. A secondary donkey-anti-rabbit antibody conjugated to Cy ^tm ^3 (Jackson Laboratories, West Grove, PA) was applied at 1:200 and incubated for 45 minutes in the dark. Three washes with PBST were performed at five minute intervals and coverslips were mounted to slides and viewed using an Olympus BX41 fluorescent microscope at 570 nm.

### Statistical Analysis

All experiments were done in triplicate. All values are given as the mean ± standard deviation (SD). Statistical analysis was done using the Students t-test and Statistica version 5 computer software (StatSoft, Inc Tulsa, OK). A p-value of < 0.05 was considered significant.

## Results

### HCV core protein induces IL-8 protein and gene expression

**Figure 1:**
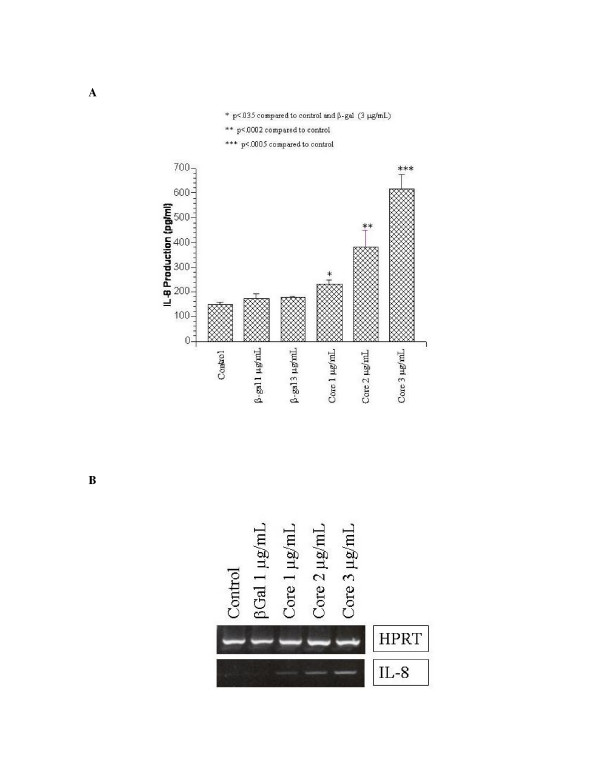
Dose-dependent increase in IL-8 expression by HCV core protein. A, NHLF were subjected to mock treatment or treatment with β-galactosidase or (β-gal) or β-galactosidase-HCV core protein (Core) at the indicated concentrations, incubated for 24 h, and supernatants assayed for IL-8 by ELISA as described in Materials and Methods. Experiments were done in triplicate. B, NHLF were subjected to mock treatment or treatment with β-galactosidase (β-gal) or β-galactosidase-HCV core protein (Core) at the indicated concentrations and incubated for 24 h. Lysates were harvested, RNA isolated and reverse transcribed, and IL-8 detected using IL-8 specific primers or HPRT as a control for RNA loading as described in Materials and Methods.

Recent studies have demonstrated that the core protein of HCV can signal extracellularly and that naked core protein is present in the serum of infected individuals [17]. To examine the role of soluble core protein in cytokine signaling in fibroblasts, we employed normal human lung fibroblasts (NHLF), which have been used as a model for cytokine expression. β-galactosidase-HCV core (1–191) fusion proteins or control β-galactosidase (β-gal) proteins were added to NHLF cultures at doses ranging from 1–3 μg/ml and incubated for 24 hours. These commercially available fusion proteins were confirmed to be endotoxin-negative and have been extensively used in studies exploring the role of HCV core in immune modulation [15,22,23]. Culture supernatants were used in an IL-8 ELISA assay (figure 1A) and cells were analyzed for IL-8 mRNA expression at 24 hours using RT-PCR, with HPRT as a control for RNA loading (figure 1B). Cells exposed to HCV core protein demonstrated significant up-regulation of both IL-8 protein and mRNA expression that was not seen in either mock- or β-gal-treated control cells as measured by ELISA. No concentration of β-gal control proteins up to 3 μg/ml elicited any IL-8 gene expression or protein production. In addition, there was no increase in expression of other cytokines, including IL-6, MCP-1, TNFα, and IL-1β, when assayed by ELISA (data not shown).

**Figure 2:**
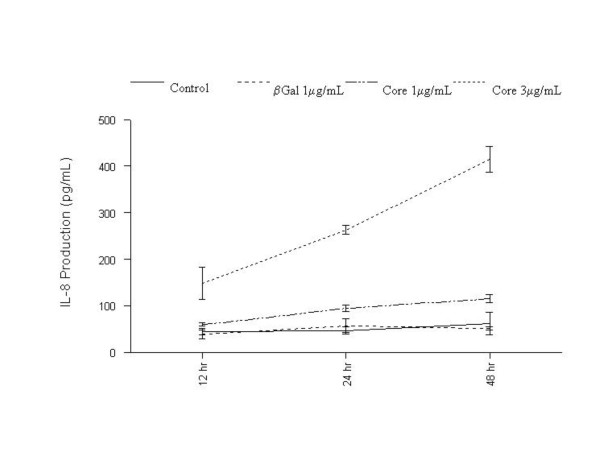
Time-dependent increase in IL-8 expression by HCV core protein. NHLF were mock-treated or treated with β-galactosidase (β-gal) or β-galactosidase-HCV core protein (Core) at the indicated concentrations. Supernatants were collected at 12, 24, and 48 h and IL-8 expression assayed by ELISA as described in Materials and Methods. All assays were done in triplicate.

To analyze the kinetics of IL-8 induction, IL-8 protein expression was determined at multiple time points following treatment of fibroblasts with HCV core protein (figure 2). Cells treated with HCV core continued to exhibit increasing levels of IL-8 production over 48 hours that were significantly and consistently elevated above mock- or β-gal-treated controls. These data suggested that HCV core significantly induced IL-8 up-regulation leading to enhanced protein expression in a dose- and time-dependent manner.

### Increased IL-8 up-regulation upon co-treatment with HCV core protein and TNFα

**Figure 3:**
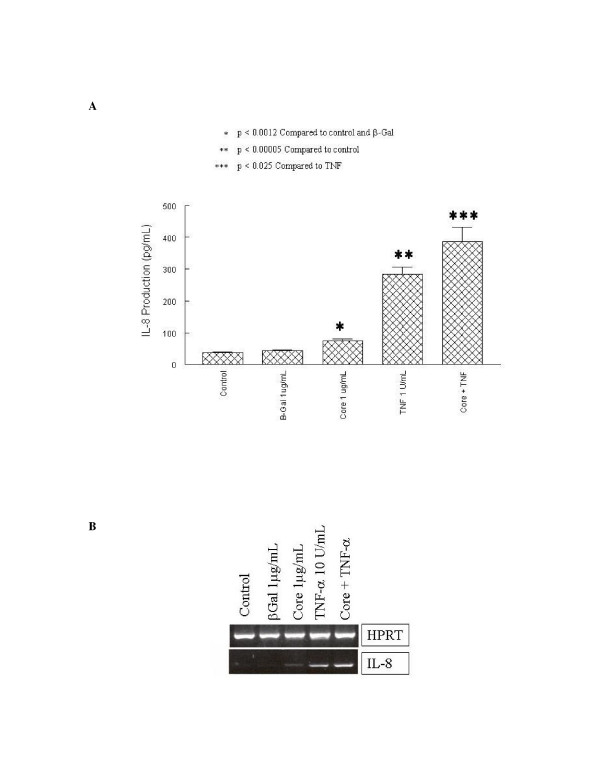
Additive increase in TNFα-induced IL-8 expression by HCV core protein. A, NHLF were subjected to mock treatment or treatment with β-galactosidase (β-gal), β-galactosidase-HCV core protein (Core), TNFα, or Core and TNFα at the indicated concentrations and incubated for 24 h. Supernatants were assayed for IL-8 by ELISA as described in Materials and Methods. Experiments were done in triplicate. B, NHLF were treated to the above conditions and incubated for 24 h. Lysates were harvested, RNA isolated and reverse transcribed, and IL-8 detected using IL-8 specific primers or HPRT as a control for RNA loading as described in Materials and Methods.

Intrahepatic levels of TNFα and IL-8 have been shown to be elevated in individuals with chronic hepatitis C infection [11]. Several investigators have suggested that HCV core protein might modulate or perhaps mimic TNFR1 signaling [24,25] and core protein has been shown to bind the cytoplasmic domain of TNFR1 [16]. Since TNFα is a strong inducer of IL-8 signaling, we wanted to determine how HCV core stimulus interacts with concomitant TNFα signaling. To accomplish this, IL-8 protein and mRNA expression were assayed in NHLF cells co-cultured with HCV core protein and TNFα (figure 3).

In these experiments, individual TNFα treatment and HCV core treatments, as expected, led to significant increases in IL-8 induction as measured by both protein (figure 3A) and gene expression (figure 3B). In cells co-treated with both TNFα and HCV core protein, however, there was an additive increase in IL-8 induction beyond what would be expected if core was mimicking TNFα and signaling only through TNFR1. The addition of antibody to TNFα to cells treated with core protein did not inhibit core-mediated IL-8 expression (data not shown).

### HCV core-mediated IL-8 secretion is dependent upon p38 phosphorylation

IL-8 signaling is a complex set of events that involves activation of several MAPK members, including most notably NFκB but also variably ERK and JNK. Efficient IL-8 signaling appears to require activation by transcription factors, such as NFκB, as well as stabilization of the resulting mRNA by p38 signaling. Because our data demonstrated a significant induction of IL-8 gene expression and protein production upon HCV core treatment, we wanted to determine if elements of these signaling pathways were being activated.

**Figure 4:**
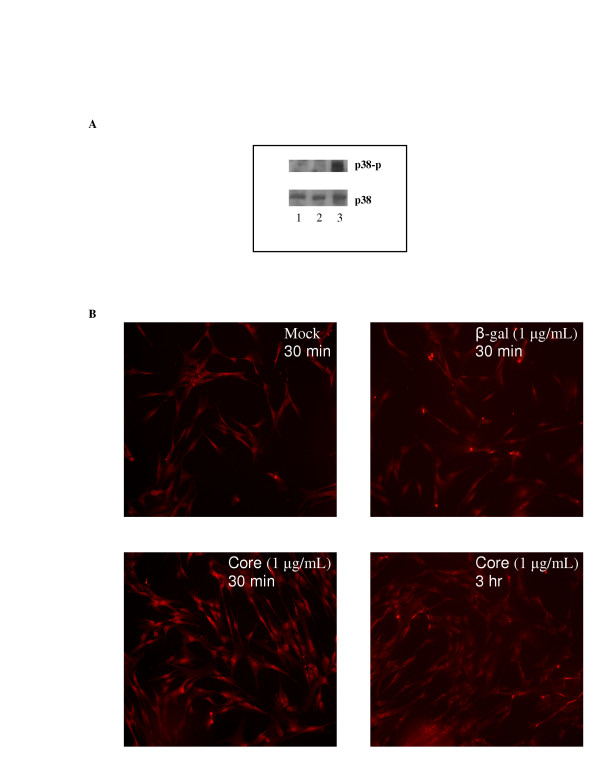
Phosphorylation of p38 in response to HCV core protein. A, Western blot analysis of human fibroblasts. NHLF were subjected to mock treatment (lane 1) or treatment with β-galactosidase (1 μg/ml) (lane 2) or β-galactosidase-HCV core protein (1 μg/ml) (lane 3) and incubated for 2 hours. Whole cell lysates of NHLF were subjected to SDS-PAGE and immunoblotted with antibodies specific for either p38 or phosphorylated p38 (p38-p) as indicated. B, Immunofluorescent staining of NHLF (40×). NHLF cultured on coverslips were subjected to mock treatment or treatment with β-galactosidase (β-gal) or β-galactosidase-HCV core protein (Core) at the indicated concentrations and incubated for 30 min or 3 hr as indicated. Cells were fixed with methanol:acetone (1:1) prior to immunofluorescent staining using an antibody to phosphorylated 38 and a secondary Cy ^tm^3-conjugated antibody. Cells were viewed and photographed using an Olympus BX41 microscope at 570 nm.

p38 plays a key role in effective IL-8 responses. [13] To examine the role of p38 signaling in HCV core-mediated responses, NHLF cells were either mock-treated or treated with β-gal or HCV core and incubated for two hours. Cells were harvested, lysed, and analyzed by immunoblotting using antibodies specific for both the native and the phosphorylated forms of p38 (figure 4A). These experiments demonstrated phosphorylation of p38 upon treatment with HCV core protein.

To confirm these findings, NHLF cells were again mock-treated or treated with β-gal or HCV core protein and incubated for 30 minutes to three hours. Cells were fixed and immunostained with antibody to phosphorylated forms of p38 and visualized by fluorescent microscopy (figure 4B). We observed nuclear localization of phosphorylated p38 consistent with activation upon treatment with core at 30 minutes. A washout effect was noted, with diminished signaling within the nucleus evident by three hours.

**Figure 5:**
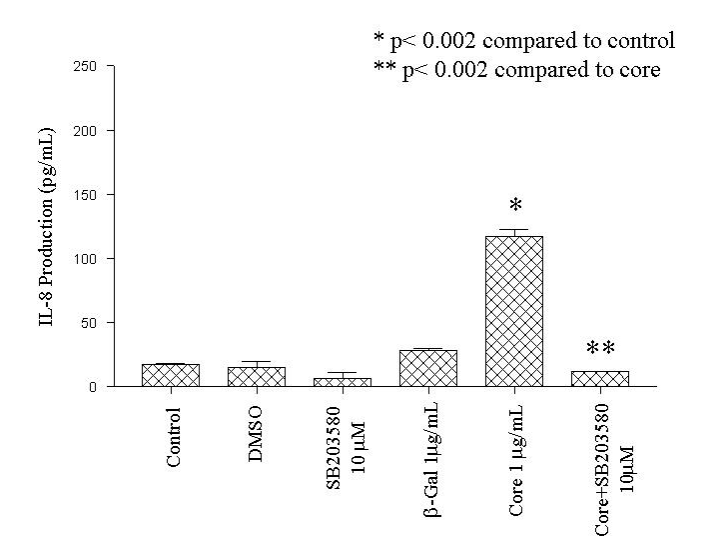
Inhibition of HCV-core induced IL-8 expression by SB203580. NHLF were either mock-treated or treated with DMSO vehicle, SB203580, β-galactosidase (β-gal), β-galactosidase-HCV core protein (Core), or Core and SB203580 at the indicated concentrations and incubated for 24 hr. IL-8 protein expression was assayed by ELISA as described above.

Because HCV core was associated with both increased IL-8 and p38 signaling, we assayed the ability of the p38 inhibitor SB203580 to inhibit overall IL-8 protein expression in these cells (figure 5). NHLF were mock-treated or treated with β-gal or HCV core protein, with or without the addition of SB203580, and incubated for 24 hours. IL-8 protein expression was determined by ELISA. These experiments demonstrated up-regulation of IL-8 protein expression by HCV core that was completely inhibited by the addition of SB203580, and suggested that p38 signaling is necessary for optimal IL-8 expression induced by HCV core protein.

### HCV core-induced IL-8 expression is dependent upon gC1qR

**Figure 6:**
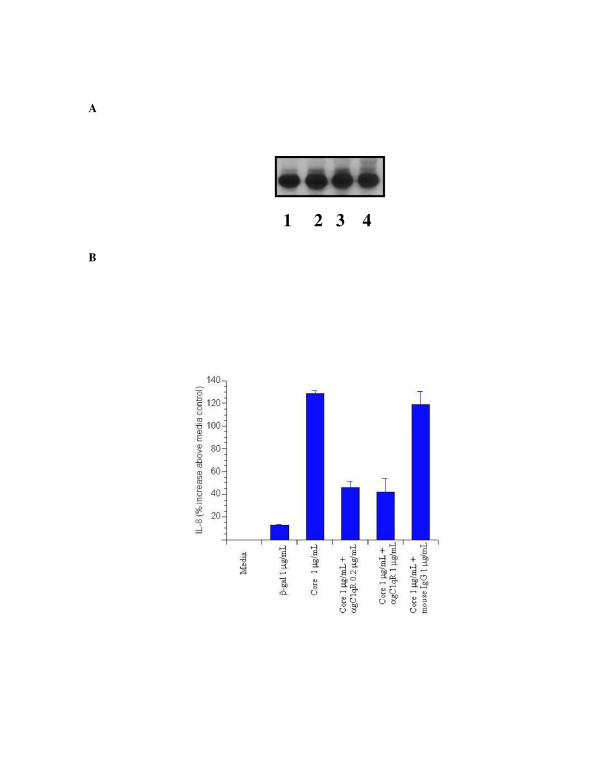
Inhibition of HCV-core induced IL-8 expression by antibody to gC1qR. A. gC1qR expression in NHLF. NHLF were mock-treated (1) or treated with β-galactosidase (2), β-galactosidase-HCV core protein (3), or β-galactosidase-HCV core protein and TNFα (4) and incubated for 24 h. Whole cell lysates of NHLF were subjected to SDS-PAGE, immunoblotted with antibodies specific for gC1qR, and visualized using ECL. A 33 kD protein was identified. B. Inhibition of HCV core-induced IL-8 expression by gC1qR. NHLF were mock-treated (Mock) or treated with β-galactosidase (β-gal), β-galactosidase-HCV core protein (Core), or β-galactosidase-HCV core protein with either antibody to gC1qR at the indicated concentrations or mouse IgG at 1 μg/ml. Supernatants were collected at 24 h and IL-8 expression assayed by ELISA as described in Materials and Methods. All assays were done in triplicate.

Recent investigations into HCV core protein have demonstrated that soluble core interacts with the complement receptor, gC1q and alters intracellular signals including MAPK. Because soluble HCV core affected the p38 MAPK signaling pathway, we wanted to assess whether this was occurring via a gC1qR-dependent process in a manner similar to experiments done in lymphocytes. Expression of gC1qR on pulmonary fibroblasts was confirmed by immunoblotting with antibody to gC1qR (figure 6A). Notably, treatment with β-gal, HCV core protein, or TNFα did not alter receptor expression level.

NHLF were subjected to treatment with β-gal, HCV core protein, HCV core protein with anti-gC1qR antibody, or HCV core protein with control isotypic antibody and IL-8 expression was assayed by ELISA (figure 6B). As in our previous experiments, HCV core induced IL-8 expression, and this was not affected by addition of murine IgG antibody. Induction of IL-8 was, however, partially blocked by antibody to gC1qR at two different doses. These data suggest that IL-8 expression induced by HCV core protein is at least partially dependent upon signaling via gC1qR.

## Discussion

The association of HCV infection with declines in pulmonary function has only recently been recognized in clinical studies. Our study represents a first attempt to examine the pathogenesis of this process. In this study, we demonstrate that the core nucleocapsid protein of hepatitis C virus induces the up-regulation of a key inflammatory cytokine, IL-8, in pulmonary fibroblasts. Treatment with soluble HCV core antigen led to increases in both IL-8 message and protein that augmented TNFα-induced IL-8 expression. This core-induced up-regulation was associated with p38 activation, which was required for core-induced signaling. IL-8 up-regulation was also dependent upon gC1qR, a known receptor for soluble HCV core.

It is important to note that our studies involved direct extracellular delivery of HCV core protein. The vast majority of studies involving HCV core have employed transfection techniques to deliver HCV proteins intracellularly, with the assumption that this would mimic viral infection of the given cell being studied. Using these techniques, we and other investigators have noted intracellular interactions with various immunomodulatory receptors, including Fas[14], TNFR [16], and LTβR[26], mediated in general through the cytoplasmic domains of those receptors.

Several studies however have now reported that nanomolar amounts of core protein are detected in the circulating blood of HCV-infected patients [17,27,28], and that core protein is secreted from transfected cell lines [29]. This has raised the possibility that HCV core can function extracellularly as a signaling antigen as a means of modulating immune responses. Recent studies focusing on the interaction of soluble core antigen with gC1qR have provided exciting and novel mechanisms by which HCV core might be exerting its immunomodulatory effects [30]. As noted by other investigators, the amounts secreted from transfected cell lines are similar to those employed in our and other investigators' studies of soluble core protein [15,19].

Our findings that HCV core antigen activates MAPK pathways involved in cytokine signaling is supported by multiple previous investigations. In a tetracycline-regulated system used to express HCV core in HepG2 cells, core expression led to activation of ERK, JNK, and p38 pathways and to an increase in cellular proliferation [31]. Similarly, studies have shown that stable transfection of HCV core results in activation of JNK and AP-1 [32]. Upon co-tranfection of HCV proteins and an IL-8 reporter plasmid into mammalian cells, HCV core exhibited the strongest effect on intracellular signaling pathways and activated the IL-8 promoter via NFκB and AP-1 [18]. These studies, however, focused primarily on signaling pathways and promoter activity rather than gene expression *per se*. Our data demonstrate that activation of at least some of these pathways does ultimately result in detectable gene and protein expression in pulmonary fibroblasts.

It is notable that multiple other HCV gene products have been associated with IL-8 upregulation, including HCV E2 [33], NS4A and 4B [34], and NS5A [9,35]. These studies were performed primarily in hepatocytyes or HeLa cells, but the effect of these gene products on pulmonary fibroblast signaling is yet to be examined. It is certainly feasible that multiple HCV proteins contribute to chemokine upregulation and inflammation in pulmonary fibroblasts, and this possibility should be the focus of future studies.

Our experiments suggest that the ability of HCV core to up-regulate IL-8 expression may be dependent upon p38 phosphorylation. This is perhaps not surprising given the putative role for p38 in stabilizing mRNA following activation of IL-8 at the transcriptional level. In current models of IL-8 signaling, p38 activation is necessary for maximal IL-8 production following stimulation [13]. Our studies would suggest that HCV core provides activation of p38 signaling, which is associated with robust IL-8 up-regulation in multiple cell types [12,36]. We cannot at this point rule out that other MAPKs, such as JNK and perhaps ERK, are not also involved in the HCV core-mediated up-regulation of IL-8. These studies are ongoing in our laboratory.

Soluble core antigen has been shown to inhibit human T cell responses via the complement receptor, gC1qR, which interestingly involved an inhibition of the ERK/MEK MAPK signaling pathway in these T cells [15,19,22]. HCV core can directly and extracellularly bind gC1qR, a phenomenon which is saturable at core concentrations of 3 μg/ml [19]. gC1qR is expressed on pulmonary fibroblasts, and our data suggest that IL-8 up-regulation by soluble HCV core in NHLF is at least partially dependent upon this receptor. It is notable that a similar induction of IL-8 expression via gC1qR was observed in HUVEC endothelial cells and was also mediated through MAPK-dependent processes [37]. Ongoing studies are examining the effect of blocking gC1qR on NFκB signaling and on MAPK signals such as p38, JNK, and ERK.

Notably, IL-8 has been found to be a key mediator of pulmonary inflammation and reactive airway disease [12]. Patients with persistent asthma have an influx of neutrophils and increased local pulmonary IL-8 levels [38]. Because IL-8 has been shown to directly provoke bronchoconstriction [39], it presumably contributes to the establishment of chronic reactive airway disease directly and indirectly by stimulating neutrophil recruitment and activation. Activation of transcription factors or kinase pathways leading to up-regulation of IL-8 expression has been implicated in the pathogenesis of multiple other viral infections, including adenovirus (ERK), CMV (NFκB), and KSHV (p38, JNK) [12].

The pro-inflammatory characteristics of HCV core protein described in our studies may be of key importance to clinical infection. Individuals with chronic HCV infection have evidence of upregulated IL-8 responses, including increased levels of IL-8 mRNA in liver biopsies from infected patients [8,10,11] and increased IL-8 protein detectable in serum compared to controls [8,9](unpublished observations, JPM.) Notably, intrahepatic IL-8 mRNA levels have correlated with both hepatic fibrosis and inflammatory indices and were associated with resistance to interferon therapy [9,11].

It is possible that a similar phenomenon is occurring in pulmonary tissue in response to HCV core and contributes to the declines in pulmonary function associated with active HCV infection [4,5]. In such a model, circulating core antigen would bind to gC1qR displayed on the surface of pulmonary fibroblasts and trigger the phosphorylation/activation of p38, NFκB, and possibly other MAPK mediators. This would lead to enhanced IL-8 gene transcription and protein expression, increased neutrophil recruitment at the local level, and ultimately the deterioration in pulmonary function observed in HCV-infected patients with lung disease [4,5,7]. It is noteworthy that patients with HCV infection even in the absence of pulmonary symptoms have been found to have increased numbers of neutrophils in bronchoalveolar fluid samples [7].

## Conclusion

Our studies point to a role for HCV core protein in up-regulating IL-8 mRNA and protein expression in a p38- and gC1qR-dependent manner and support much of the growing body of literature that suggests that HCV core is pro-inflammatory in specific cells. Further dissection of the pathways involved in HCV core-mediated signaling may provide a clearer understanding of the pathogenesis of pulmonary and hepatic disease in HCV-infected individuals and provide targets for modulating its effects.

## Abbreviations

HCV: hepatitis C virus

NHLF: normal human lung fibroblasts

IL-8: interleukin 8

MAPK: mitogen-activated protein kinase

BALF: Bronchoalveolar lavage fluid

EBV, Epstein-Barr virus

IFN, interferon

LPS, lipopolysaccharide

CMV: cytomegalovirus

KSHV: Kaposi's sarcoma-associated herpes virus

ELISA, enzyme-linked immunosorbent assay

DLCO: diffusing capacity for carbon monoxide

FEV1: forced expiratory volume at one second

COPD: chronic obstructive pulmonary disease

JNK: jun-kinase

NFκB: nuclear factor kappa B

TNFR: tumor necrosis factor receptor

HPRT: hypoxanthine phosphoribosyltransferase

PMSF: phenylmethylsulfonyl fluoride

PAGE: polyacrylamide gel electrophoresis

## Competing interests

The author(s) declare that they have no competing interests.

## Authors' contributions

**JPM **conceived the study, participated in its design, coordinated all experiments and drafted the manuscript. **SMF **carried out the IL-8 immunoassays and RT-PCR reactions and helped draft the manuscript. **DCP **completed all immunofluorescence studies. **SAL **carried out experiments involving TNFα including immunoassays. **DSC **participated in study design and data interpretation and provided critical manuscript review. **GK **participated in the study design, coordinated experiments, and drafted the manuscript in coordination with JPM. All authors read and approved the final manuscript.
